# Sputum matrix metalloproteinase-9 is associated with the degree of emphysema on computed tomography in COPD

**DOI:** 10.1186/2213-0802-1-11

**Published:** 2013-06-06

**Authors:** Rekha Chaudhuri, Charles McSharry, Mark Spears, Jeffrey Brady, Christal Grierson, C Martina Messow, Gino Miele, Karl Nocka, William MacNee, Martin Connell, John T Murchison, Michael Sproule, Omar Hilmi, Douglas K Miller, Neil C Thomson

**Affiliations:** 1 Immunology, Institute of Infection, Immunity & Inflammation, University of Glasgow and Gartnavel General Hospital, Glasgow, Scotland G12 OYN UK; 2 Pfizer Research/Translational Medicine Research Collaboration, Dundee, UK; 3 Pfizer Research/Translational Medicine Research Collaboration, Cambridge, MA USA; 4 Pfizer Research/Translational Medicine Research Collaboration, Collegeville, PA USA; 5 Robertson Centre for Biostatistics, University of Glasgow, Glasgow, UK; 6 MRC Centre for Inflammation Research, Medical Physics and Clinical Radiology, University of Edinburgh, Edinburgh, UK; 7 Radiology Department, Gartnavel General Hospital, Glasgow, UK; 8 Ear Nose and Throat Department, Gartnavel General Hospital, Glasgow, UK

**Keywords:** MMP-9, *MMP-9* expression, COPD, Computed tomography

## Abstract

**Background:**

Matrix-metalloproteinase (MMP)-9 has been implicated in the pathogenesis of COPD, although its link to disease severity is unclear. The purpose of the study was to examine the relationship between disease severity assessed by lung function and computed tomography (CT) and sputum MMP-9 expression, concentration and activity in patients with COPD.

**Findings:**

In 53 COPD subjects, smokers and ex-smokers; 46 healthy controls, smokers and never smokers, we measured sputum MMP-9 concentrations (ELISA) and enzyme activity (FRET), sputum *MMP-9* mRNA expression, spirometry, diffusing capacity for carbon monoxide (DLco) and CT assessment of emphysema (% low attenuation areas below-950 Hounsfield units).

Sputum MMP-9 concentrations and mRNA expression in COPD subjects were significantly greater than in healthy never-smokers (p = 0.007 and p = 0.001 respectively) and similar to those in healthy smokers. Disease severity when assessed by the extent of emphysema measured by CT, but not by spirometry or DLco values, was directly associated with sputum MMP-9 concentrations [r = 0.442 (0.171, 0.634), p = 0.020], and MMP-9 activity [r = 0.447 (0.219, 0.643), p = 0.010]. In moderate to severe COPD, increased *MMP-9* mRNA expression levels were associated with reduced post-bronchodilator FEV_1_ [r = −0.530 (−0.686, -0.327), p < 0.001], FEV_1_/FVC ratio [r = −0.551 (−0.701, -0.354), p < 0.001] and reduced DLco [r = −0.399 (−539, -0.102), p = 0.048].

**Conclusions:**

Sputum MMP-9 concentrations in COPD are directly associated with the extent of emphysema measured by CT and *MMP-9* expression levels are inversely associated with DLco. These findings support a role for MMP-9 in the pathogenesis of COPD.

**Electronic supplementary material:**

The online version of this article (doi:10.1186/2213-0802-1-11) contains supplementary material, which is available to authorized users.

## Findings

Inappropriate expression and excessive activity of several matrix metalloproteinases (MMP), including MMP-9 [1, 2] has been implicated in the tissue destructive processes associated with chronic lung diseases including COPD [2–7]. Increased sputum MMP-9 protein concentrations and/or activity are reported in current smokers with COPD compared to healthy smokers [8, 9]. However, a definitive role of MMP-9 in the development of emphysema is uncertain. For example, mice deficient in MMP-9 develop the same degree of cigarette smoke-induced inflammation and airspace enlargement as control mice and human macrophages *MMP-9* mRNA is present in areas of lung irrespective of the presence of emphysema [10]. Nevertheless circulating monocytes from subjects with advanced emphysema on computed tomography (CT) produced more MMP-9 than controls [10]. In previous clinical studies there is a lack of information about concentrations, activities and expression of MMP-9 in well characterized patients with COPD and it is unknown whether MMP-9 activity is linked to disease severity. The aim of the study was to test the hypothesis that MMP-9 concentrations and activity in COPD are associated with disease severity as assessed by lung function and computed tomography.

### Methods

#### Subjects

A cross sectional study was performed in subjects with COPD and healthy controls as part of the Glasgow COPD and Asthma Biomarker study [7]. Clinical, physiological and immunological plus imaging (COPD groups only) measurements were performed at baseline. Participants were recruited with mild, moderate and severe COPD (GOLD stages I (n = 14), II (n = 23) and III (n = 16) [11]; (both current smokers and ex-smokers), and healthy smokers and never smokers, defined as volunteers with no known respiratory disease, no chronic respiratory symptoms and normal spirometry.

The West Glasgow Research Ethics Committee approved the study and all patients gave written informed consent.

#### Measurements

Spirometry was performed according to American Thoracic Society guidelines [12]. Lung volumes and diffusing capacity for carbon monoxide (DLco) were performed using the body box technique (Zan500 Body Plethysmography, nSpire Health Limited, Hertford, UK). Sputum induction was performed as previously described [7]. CT scans of the Chest were performed at full inspiration using 16 slice Brightspeed and 64 slice Lightspeed (GE CT scanner, Milwaukee, Wisconsin, USA) with the following parameters: 120 KV, 100 mAs, collimation 1mm, reconstruction slice thickness 0.65mm, reconstruction slice separation 0.5mm, pitch of 1 and the data was reconstructed with a CHST filter. All scans were evaluated centrally at the University of Edinburgh. Emphysema was quantified as the percentage of lung CT voxels below a threshold of −950 Hounsfield Units-% low attenuation areas (LAA)-950, using the software Pulmonary Workstation 2.0 (VIDA Diagnostics, Iowa City, IA, USA) as described previously [13–15]. MMP-9 ELISA and MMP-9 activity kits (fluorescence resonance energy transfer (FRET) method) were supplied by R&D Systems Europe Ltd. (Cat. Nos. DMP900 and F9M00 respectively; Abingdon, UK). Assays were performed in accordance with the instructions provided; however the activity assay was read kinetically over 4 hours as this showed better reproducibility than an end-point read-out. Samples were not chemically activated with amino-phenyl mercuric acetate to allow estimation of endogenous enzyme activity. The MMP-9 assays were validated for sputum. Samples spiked with exogenous MMP-9 (Lot#40011-70; Wyeth, USA) showed bias of <11% with 101-110% recovery; inter-assay CV was <8.8% and intra-assay CV was <7.9%; concentrations were stable up to 5 freeze-thaw cycles. Sputum cell *MMP-9* expression levels (Affymetrix number 203936_s_at, on U133 + 2 chips) were measured in RNA isolated from airway inflammatory infiltrate cells harvested from induced sputum in healthy never smokers (n = 15), healthy smokers (n = 13), smokers with moderate to severe COPD (n = 15) and ex-smokers with moderate to severe COPD (n = 12). Expression levels were compared after global normalisation.

#### Analysis

Variables were summarised as median (inter-quartile range). Values were compared between patient subgroups using two-sample Wilcoxon tests or Kruskal-Wallis test. Associations of MMP-9 with biomarkers were assessed using Spearman rank correlation coefficient with bootstrap 95% confidence intervals. All analyses were carried out in R version 2.11.0 [16].

### Results

#### Baseline demographics

Demographics and baseline characteristics are previously reported [7]. Briefly, median (IQR) age [years] of COPD volunteers was 65 (61.2, 68.9), healthy never smokers 49.9 (42.1, 55.8) and healthy smokers 53.3 (45.1, 57.8). In healthy never smokers FEV_1_% predicted median (IQR) was 105.5 (95.2, 115.0) and in healthy smokers this was 92.5 (87.5, 100.2). Lung function in the COPD group was as follows: median (IQR) post-bronchodilator FEV_1_% predicted, 65.0 (48.0-80.0); post-bronchodilator FEV_1_/FVC ratio, 64.0 (50.0-67.0); DL_CO_% predicted, 60.0 (47.5-70.0).

#### Sputum MMP-9 concentrations and activity

**Figure 1:**
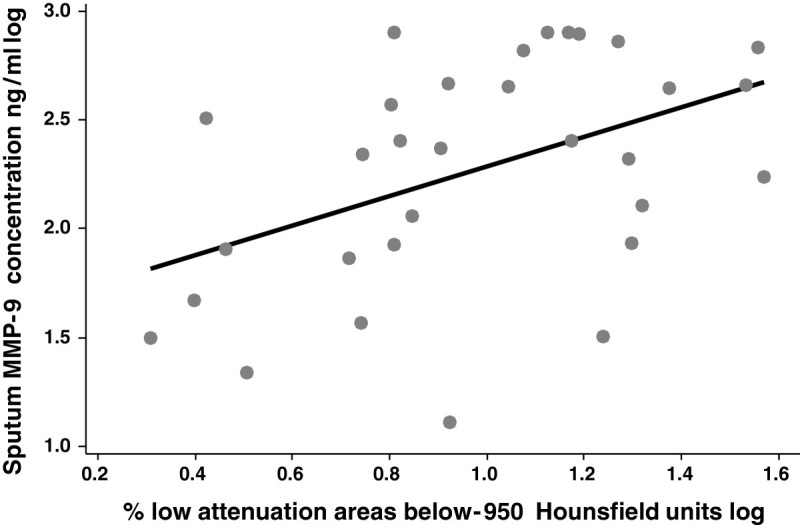
**Correlation of sputum MMP-9 concentration (ng/ml) with computed tomography measure of emphysema [the low attenuation areas below −950 Hounsfield Units taken as an index of emphysema (% LAA −950) [r = 0.442 (0.171, 0.634), p = 0.020)].**

Sputum MMP-9 concentrations were increased in COPD subjects compared to healthy never smokers (p = 0.007), but were similar to healthy smokers (p = 0.343) (Table 1). Sputum MMP-9 enzyme activity was increased in ex-smokers with COPD compared to healthy never smokers (p < 0.001). Ex-smokers with COPD had higher concentrations and activity of sputum MMP-9 than current smokers with COPD (p = 0.002 and p < 0.001 respectively). Sputum MMP-9 concentrations and activity did not differ significantly between mild, moderate and severe COPD groups (data not shown). The MMP-9 protein concentration and activity did not correlate significantly with FEV_1_ or DLco% predicted. The extent of emphysema (% LAA −950) was significantly associated with sputum MMP-9 protein concentration [r = 0.442 (0.171, 0.634), p = 0.020,)] (Figure 1) and MMP-9 activity [r = 0.447 (0.219, 0.643), p = 0.010]. Sputum MMP-9 protein concentration, r = 0.724 (0.557, 0.839), p < 0.001,)] and MMP-9 activity, r = 0.682 (0.520, 0.800), p < 0.001, were associated with the percentage of sputum neutrophils. The sputum MMP-9 protein concentration correlated with sputum cell *MMP-9* mRNA expression [r = 0.510 (0.292, 0.664), p < 0.001].

**Table 1:** S**putum MMP-9 concentrations and activity in sputum fluid from patients with COPD and healthy controls [Median (IQR)]**

		MMP-9 (ng/mL)	MMP-9 activity (ng/mL)
**Healthy Control**	**All**	49.80 (26.35, 136.50)	18.90 (12.50, 38.35)
	**Never smoker**	34.05 (24.45, 131.00)	16.60 (12.50, 28.42)
	**Smoker**	69.00 (38.65, 184.00)	18.90 (13.40, 46.45)
	p value ^1^	p = 0.161	p = 0.285
**COPD**	**All**	137.50 (45.52, 362.95)	26.65 (12.50, 51.85)
	**Ex- smoker**	214.75 (101.12, 445.05)	42.10 (26.02, 116.10)
	**Smoker**	56.10 (28.90, 190.70)	12.50 (12.50, 32.52)
	p value ^2^	**p = 0.002**	**p < 0.001**
	p value ^3^	**p = 0.007**	p = 0.187
	p value ^4^	p = 0.343	p = 0.787
	p value ^5^	**p < 0.001**	**p < 0.001**
	p value ^6^	p = 0.617	p = 0.086

^1^ Healthy smokers vs. healthy never smoker; ^2^ COPD smokers vs COPD ex-smokers;

^3^ All COPD subjects vs. healthy never smokers; ^4^ All COPD subjects vs. healthy smokers;

^5^ COPD ex-smokers vs. healthy never smokers; ^6^ COPD smokers vs. healthy smokers.

#### Sputum MMP-9 mRNA expression

**Figure 2:**
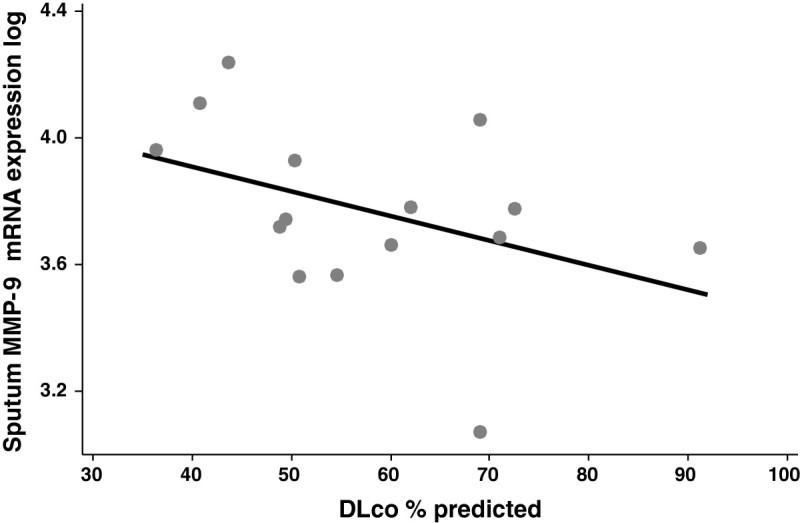
**Correlation of sputum** ***MMP-9*** **mRNA expression and diffusing capacity for carbon monoxide (DLco) % predicted [r = −0.399 (−0.539, -0.102), p = 0.048].**

Sputum cell *MMP-9* expression levels [median (IQR)] normalized fluorescent intensity units]) were significantly greater in ex-smokers with COPD, [4622 (3161, 5962)], current smokers with COPD, [5085 (4378, 9338)] and healthy smokers, [4111 (1623, 6202)] compared with healthy never smokers, [1650 (1023, 3443)]; (p = 0.001 for each). Sputum *MMP-9* expression levels correlated negatively with post-bronchodilator FEV_1_% predicted [r = −0.530, (95% CI −0.686, -0.327), p < 0.001], post-bronchodilator FEV_1_/FVC [(r = −0.551 (−0.701, -0.354], p < 0.001], and DLco [r = −0.399 (−0.539, -0.102), p = 0.048], (Figure 2) but were not significantly associated with the CT extent of emphysema [r = 0.266 (−0.020, 0.446), p = 0.115].

### Discussion

This study found that sputum MMP-9 concentrations and mRNA expression in COPD are significantly greater than in healthy never-smokers, that sputum MMP-9 concentrations are directly associated with the extent of emphysema measured by CT and that higher *MMP-9* expression levels are associated with greater airflow obstruction and reduced diffusing capacity. Taken together, these findings provide indirect evidence for a link between MMP-9 and disease severity in COPD.

Previous studies have reported increased MMP-9 protein concentrations and/or activity in current smokers with COPD compared to healthy smokers [8, 9]. In contrast to the finding from these studies we found that sputum MMP-9 concentrations and activity in COPD subjects were similar to healthy smokers. There are differences in the severity of disease and smoking status between these studies. The patients with COPD studied previously [8, 9] had in general more severe disease compared to the participants in our study, which could explain the different results [17]. Both current and ex-smokers with COPD were recruited to the present study, whereas previous studies included only current smokers with COPD [8, 9]. Smoking status is however unlikely to explain the different results as ex-smokers had higher MMP-9 levels and activity than current smokers with COPD. The ex-smokers with COPD in the present study had more severe disease, as assessed by the extent of emphysema on CT, which may explain the higher sputum MMP-9 concentrations in this group.

The severity of COPD, when assessed by the extent of emphysema on CT scanning, but not by spirometry values, was significantly associated with sputum MMP-9 concentrations and activity. We found no significant association between sputum MMP-9 levels and DLco; although the extent of emphysema measured by CT often correlates with DLco in COPD, this association is not always found. The findings of an association of greater *MMP-9* expression in moderate to severe disease with greater airflow obstruction and reduced diffusing capacity as well as more extensive emphysema on CT scanning with MMP-9 concentrations suggests a role for MMP-9 in the pathogenesis of emphysema. Alternatively, increased sputum MMP-9 may be a marker of disease severity in patients who already have emphysema. Long-term clinical trials of MMP-9 inhibitors in COPD would help establish whether blocking MMP-9 activity attenuates the development and progression of emphysema. Previously, we reported that sputum MMP-12 concentrations and activity in patients with COPD are directly associated with the extent of emphysema measured by CT [7] suggesting that blocking both MMP-9 and MMP-12 may be more effective than inhibiting either MMP-9 or MMP-12 alone.

In conclusion, disease severity in COPD when assessed by the extent of emphysema measured by CT is directly associated with sputum MMP-9 concentrations. Interventions directed at inhibiting MMP-9 activity, either alone or in combination with MMP-12 blockers, should be investigated for their potential to attenuate the development or progression of emphysema.

## Authors’ original submitted files for images

Below are the links to the authors’ original submitted files for images. Authors’ original file for figure 1
Authors’ original file for figure 2
