# Supplementary figures and images for: Sputum matrix metalloproteinase-9 is associated with the degree of emphysema on computed tomography in COPD
Source: Transl Respir Med. 2013 Jun 6;1:11. doi: 10.1186/2213-0802-1-11 (PMC6733425; doi:10.1186/2213-0802-1-11)

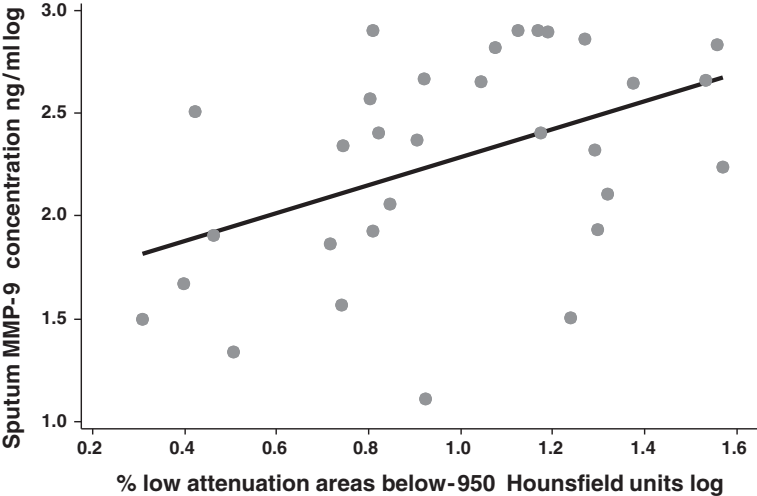

Supplement: Supplementary file 1 — Authors’ original file for figure 1 [file 40247_2013_11_MOESM1_ESM.pdf]

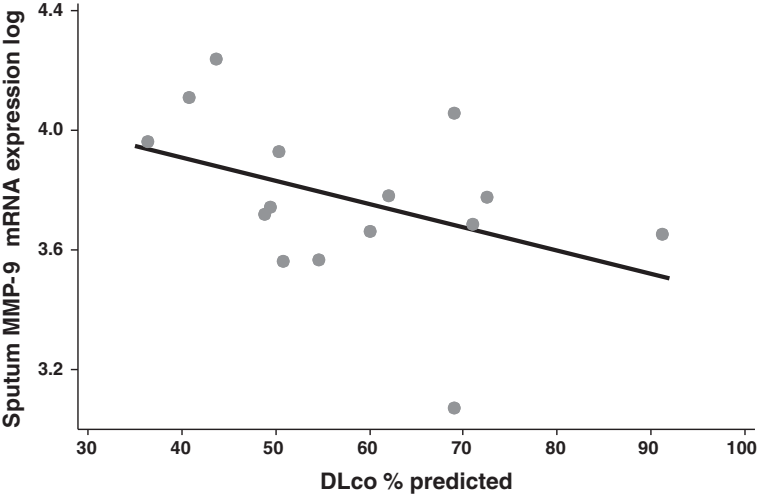

Supplement: Supplementary file 2 — Authors’ original file for figure 2 [file 40247_2013_11_MOESM2_ESM.pdf]
